# Community-based interventions addressing open defecation in Sub-Saharan Africa: a scoping review

**DOI:** 10.1080/16549716.2026.2655033

**Published:** 2026-04-13

**Authors:** Ami Makita, Natalia Calanzani

**Affiliations:** a Institute of Applied Health Sciences, University of Aberdeen, Aberdeen, UK; b Department of Healthcare Quality Assessment, University of Tokyo, Tokyo, Japan

**Keywords:** Open defecation, Community-Led Total Sanitation, CLTS, community-based interventions, WASH

## Abstract

Open defecation (OD) remains a major public health issue, particularly in rural Sub-Saharan Africa (SSA), where a significant proportion of the population still lacks access to basic sanitation. It increases the risk of infectious diseases, psychosocial stress, and sexual violence, especially for women. Community-based interventions, such as Community-Led Total Sanitation (CLTS), have been implemented to eliminate OD through behaviour change. This scoping review aimed to map community-based approaches used to eliminate OD in SSA. It followed Joanna Briggs Institute (JBI) and PRISMA-ScR guidelines. Peer-reviewed primary studies published since 2010 on community-based interventions addressing OD in SSA were included. Nationwide or poorly described interventions were excluded. Searches were conducted in MEDLINE, Web of Science, and CINAHL. Two reviewers independently screened, extracted, and synthesised data. Fifteen studies on community-based interventions were included. Most (*n* = 12) focused on CLTS, with some combining additional components or using alternative interventions including health education, or shared latrine construction. Outcomes included latrine use (*n* = 6), OD reduction (*n* = 5), both latrine use and OD reduction (*n* = 1), diarrhoea incidence (*n* = 2), Water, Sanitation, and Hygiene (WASH) behaviours (*n* = 2), and psychosocial stress (*n* = 1). CLTS-related interventions generally reported on improved latrine ownership and reduced OD. Other non-CLTS interventions often reported limited or inconsistent improvements. Barriers included limited facilitator skills and weak implementation fidelity, while facilitators included small village size, and strong social cohesion. In conclusion, context-specific interventions, engaging leaders, and focusing on smaller communities may facilitate reduction in OD practices. Future research and practice should prioritise long-term evaluation and sustaining behaviour change.

## Paper context

**Main findings:** In Sub-Saharan Africa, Community-Led Total Sanitation was the most frequently implemented community-based intervention, and several studies reported improvements in latrine use and reductions in open defecation.

**Added knowledge:** Adding additional components to Community-Led Total Sanitation does not consistently improve outcomes, and more effective implementation requires adaptation to local customs and contextual conditions.

**Global health impact for policy and action:** This review clarifies the role of community-led interventions in achieving Sustainable Development Goal 6 on eliminating open defecation and highlights the need for future policies, programmes, and research to focus on sustainability and implementation quality.

## Background

Open defecation (OD) is a major public health issue. More than 1.5 billion people globally still lack access to basic sanitation services such as toilets, and over 419 million people continue to practice OD [1]. Although the global rate of OD has decreased over the past few decades, it remains high in rural areas of Sub-Saharan Africa (SSA), where 25% of the population still practiced it in 2022 [2]. OD poses various risks, including lack of privacy and sexual violence for women, increased risk of snake bites, and psychosocial stress when defecating at night [3]. It is also closely linked to the spread of infectious diseases and contributes to higher rates of diarrhoeal diseases and typhoid fever [1]. The elimination of OD is a core aspect of the United Nations’ Sustainable Development Goals, specifically Goal 6 which aims to ensure availability and sustainable management of water and sanitation for all [4]. Interventions implemented to eliminate OD include latrine subsidies, sanitation-related health education, and social marketing. Many interventions that aim to reduce OD and promote toilet use tend to follow community-based approaches [5,6]. A well-known example is Community-Led Total Sanitation (CLTS), which has been implemented in around 60 countries, mainly in Africa and Asia. Some countries have adopted it as part of their national sanitation strategies [7,8]. CLTS is a community-based approach targeting collective behaviour change without providing financial subsidies. It encourages cooperation and solidarity among local residents to achieve the elimination of OD [9]. Community-based interventions like CLTS are often well accepted because they can be adapted to the specific context of each community [10]. Synthesising existing evidence on such community-based interventions is essential for informing future actions in regions where OD remains prevalent.

This scoping review aims to map the community-based approaches used to eliminate OD in SSA, where OD rates remain high compared to other regions. The review seeks to address the following research questions:

1. What community-based interventions have been adopted in SSA to eliminate OD, and what are their main characteristics?
2. What are the outcomes of community-based interventions addressing OD in SSA?
3. What are the barriers and facilitators to implementing community-based interventions for OD in SSA?

## Methods

This scoping review followed the methodological guidance of the Joanna Briggs Institute [11] and the PRISMA extension for scoping reviews (PRISMA-ScR) [12]. The review protocol is available on the Open Science Framework platform [13].

### Eligibility criteria

This scoping review included peer-reviewed, primary studies published in or after 2010, the year when the United Nations General Assembly officially recognised access to safe and clean drinking water and sanitation as an essential human right for the full enjoyment of life and all other human rights [14]. Following the Population, Concept, and Context (PCC) mnemonic [11], the review included studies that described community-based (including schools) interventions aimed at addressing OD (Concept) among individuals of school age and older (Population) in countries within SSA (Context).

Interventions implemented at the community level were included, while those delivered exclusively on a nationwide scale were excluded. Only studies explicitly describing implemented interventions in the main text – including details such as the intervention facilitator, implementation period, and programme steps or phases – were included. Studies lacking such details or focusing solely on sustainability or cost-effectiveness were excluded.

Primary qualitative, quantitative, and mixed-methods peer-reviewed studies were included. Systematic reviews, scoping reviews, editorials, case reports, expert opinions, reports, and conference proceedings without full text were excluded. Articles published in English, French, and Portuguese were eligible for inclusion.

### Information sources

A literature search was conducted using Ovid MEDLINE, Web of Science, and EBSCO CINAHL. The final search was conducted on 26 June 2025.

### Search strategy

Search strategies were informed by publications about OD [15,16], then tested for accuracy and sensitivity. Both keywords and subject headings were used. The search strategies required that articles included the term ‘open defecation’ (or variations) to avoid retrieving publications on sanitation that did not explicitly address OD and were therefore ineligible for inclusion. The full search strategies for all databases are available (Appendix 1). While subject headings and search operators were adapted to meet database requirements, the core search terms were applied consistently across all of them.

### Source of evidence selection

Duplicate records across databases were identified and removed using Rayyan [17], with manual checking and confirmation. Two reviewers then independently screened the titles and abstracts for relevance. This was followed by full-text screening against eligibility criteria, conducted independently by the same two reviewers. Any discrepancies were resolved through discussion. Reasons for exclusion at the full-text screening stage were documented.

### Data extraction

Data were extracted by one reviewer (and checked by a second reviewer) into an Excel spreadsheet, including the following information: author and year, title, study aims, study design, country and region, type of intervention, intervention setting, characteristics of the intervention, target population, number of participants, sex and age, duration of the intervention, presence of a comparator group, whether outcomes were reported, type of outcome, reported barriers, and reported facilitators.

### Analysis of evidence

Results were described in text and tables, organised to answer each of the review questions. To facilitate a detailed analysis, additional tables were created about specific aspects of the interventions, outcomes, and barriers and facilitators. Intervention-related tables captured information on implementation periods, programme steps, any adaptations made, barriers, and facilitators. Outcome-related tables summarised sample sizes, respondent characteristics, data collection methods and periods, and reported outcomes. To address the heterogeneity of outcomes, the reported findings were grouped into three domains: infrastructure-related, behavioural, and health-related outcomes. These analyses allowed for a comprehensive synthesis and tabular presentation of the evidence.

## Results

**Figure 1.**
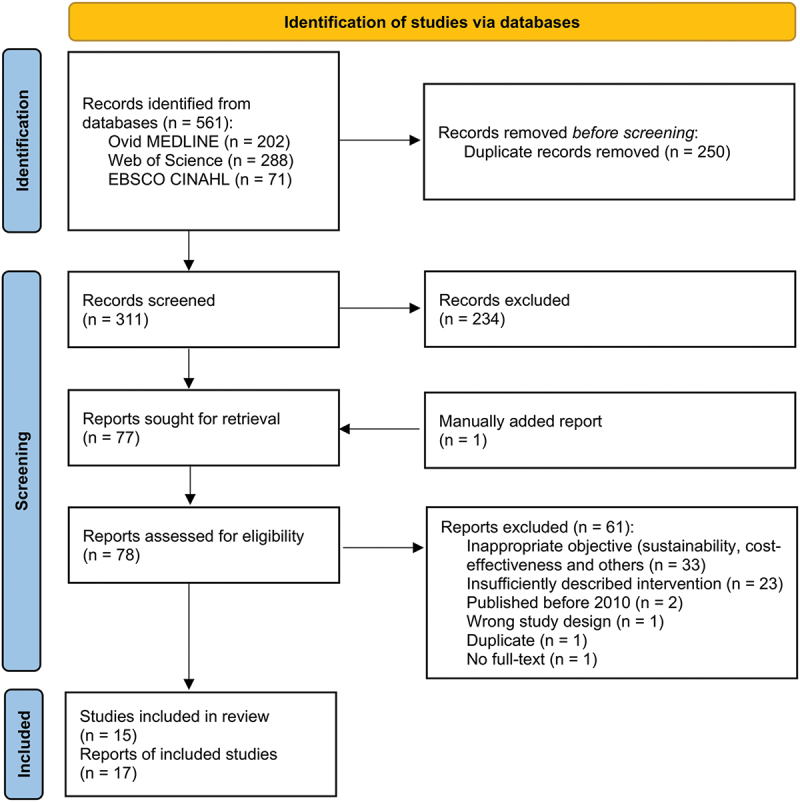
Flow diagram of screening process.

Out of the 561 records retrieved from database searches, 311 remained after duplicate removal and were screened based on titles and abstracts. Of these, 77 articles were assessed for eligibility through full-text screening. During this stage, one record was identified as a study protocol; the corresponding primary study had not been retrieved in the initial search because its abstract/subject headings lacked the necessary keywords. As a result, the primary study was manually searched, assessed for eligibility, and subsequently included. Of the 78 full-text articles assessed, 17 reports met the inclusion criteria. As two of these were study protocols, a total of 15 completed studies were included in the review (Figure 1).

In terms of overall characteristics (Table 1), all 15 studies were published in English. The study designs include seven randomised trials, two quasi experimental designs, one controlled before-and-after design, four cross-sectional studies and one case-control study. Regarding the study locations, the largest number of interventions were conducted in Ethiopia [18–21] and Ghana [22–25] (four studies each). This was followed by Mali [26,27] and Mozambique [28,29], with two interventions each. Eswatini [30], Uganda [31], and Zambia [32] each had one intervention. Most of the study areas were rural settings; however, one study focused exclusively on an informal slum in Maputo, the capital city of Mozambique [29]. The most common type of intervention was CLTS implemented on its own (eight studies) [19,22–24,26–28,31]. In three studies, CLTS was combined with additional components such as training for natural leaders (NL) and orientation sessions for community chiefs and was compared with CLTS alone [20,25,32]. One study compared CLTS with subsidy-based interventions (SBSI) [30]. Three interventions did not involve CLTS. These included the delivery of health education [18,33], the Andilaye approach [21], and the construction of shared latrines [29]. In this review, ‘latrine ownership’ refers to whether a household possessed a latrine, ‘latrine coverage’ refers to the proportion of households with access to a latrine within a defined area, and ‘latrine access’ refers to the availability of a latrine for use, including shared facilities.

**Table 1.** Summary of study characteristics.

Author (Year)	Study aim	Study design	Country	Target population and number	Type of intervention	Duration of intervention	Outcome domain(s)	Specific outcome(s)
Alemu et al (2023)	To assess the impact of information provided by Health Extension Workers^a^ (HEWs) on WASH practices.	Cross-sectional	Ethiopia	Rural area, 9 regional states and 2 city administrations	Health Extension Program^b^ (HEP)	2018–2019	Behavioural and infrastructure-related outcome	WASH practice (access to safe water, hygiene and sanitation)
Alzúa et al (2019)	To examine how CLTS influences sanitation behaviours.	Randomized Controlled Trial (RCT)	Mali	Koulikoro region, 2.4 million people	CLTS	2011–2013	Behavioural and infrastructure-related outcome	Open Defecation (OD) practice; Latrine ownership
Cha et al (2021)	To evaluate the effects of CLTS on diarrhoea rates in children.	Cluster-randomized trial (CRT)	Ethiopia	Cheha and Enemor Ena Ener districts, 212 villages	CLTS	2015–2017	Health-related outcome	Childhood diarrhoea (incidence, prevalence)
Crocker et al (2016a)	To evaluate the effect of adding natural leader^c^ (NL) training to CLTS.	Cluster-randomized field trial	Ghana	The Central, Upper West, and Volta regions, 20 villages	CLTS vs CLTS+NL training	2012–2014	Behavioural outcome	OD practice
Crocker et al (2016b)	To compare the effectiveness of HEWs-led CLTS and teacher-led CLTS on improving sanitation behaviours.	Quasi-experimental	Ethiopia	Deksis and Dara districts, 6 kebeles^d^	CLTS	2012–2013	Behavioural outcome	OD practice
Freeman et al (2022)	To assess if the Andilaye sanitation intervention affected WASH behaviours and mental health.	CRT	Ethiopia	West Gojjam and South Gondar Zones, 3 districts	Andilaye^e^	2016–2019	Behavioural outcome	WASH practice
Harter et al (2018)	To assess how CLTS participation affects latrine ownership and identify key psychosocial and contextual factors involved.	Cross-sectional	Mozambique	Nampula region Case: 26 communities Control: 6 communities	CLTS	2012–2014	Infrastructure-related outcome	Latrine ownership
Harter et al (2019a)	To identify and measure factors affecting latrine coverage and their impact on CLTS success.	Cross-sectional (embedded in RCT)	Ghana	Sawla-Tuna-Kalba, and Bole district in the Northern Region, 102 communities	CLTS	2016–2017	Infrastructure-related outcome	Latrine coverage
Harter et al (2019b)	To clarify the role that social identification plays in achieving an OD-free environment through CLTS.	Cluster-RCT	Ghana	Sawla-Tuna-Kalba, and Bole district in the Northern Region, 132 communities	CLTS	2016–2018	Behavioural outcome	OD practice
Harter et al (2020)	To investigate the effectiveness of CLTS and CLTS+data-driven and population-tailored interventions following the RANAS^f^ approach	Cluster-RCT	Ghana	Sawla-Tuna-Kalba, and Bole district in the Northern Region, 132 communities	CLTS vs CLTS + RANAS-based interventions	2016–2018	Infrastructure-related outcome	Latrine construction
Mlenga and Baraki (2015)	To compare CLTS and subsidy-based sanitation interventions (SBSI) for reducing OD and improving sanitation coverage.	Case-control	Swaziland (now Eswatini)	Four constituencies in Shiselweni and Lubombo regions Case (CLTS):100 households Control (SBSI):100 households	CLTS vs SBSI	2012–2014	Infrastructure-related outcome	Latrine coverage
Okolimong et al (2020)	To compare CLTS-implementing and non-implementing areas to assess the potential effects of CLTS.	Comparative cross-sectional	Uganda	Pallisa district, 301 villages	CLTS	2011–2018	Behavioural and infrastructure-related outcome	Open Defecation Free^g^ (ODF) status; sanitation and hygiene status
Pickering et al (2015)	To explore the effectiveness of CLTS on coverage and quality of household sanitation facility, child health, and defecation behaviour.	CRT	Mali	Koulikoro region, 402 villages	CLTS	2011–2013	Health-related outcome	Childhood diarrhoea (prevalence)
Shiras et al (2018)	To examine how improved shared latrines in informal urban areas affect sanitation-related stress and women’s well-being.	Controlled before-and-after	Mozambique	Maputo, informal slum, About 840,000. (70% of Maputo’s population)	Shared sanitation intervention	2015–2016	Health-related outcome	Psychosocial stress; user satisfaction
Tiwari et al (2017)	To examine the impact of chiefs’ engagement on community sanitation access during CLTS implementation.	Quasi-experimental design	Zambia	Rural area, Over 15,000 villages	CLTS vs CLTS+ Chiefdom orientations	2013–2015	Infrastructure-related outcome	Latrine access

^a^HEWs = Government employees responsible for delivering health services at the community level within the HEP [33].

^b^HEP = A flagship programme in the health sector implemented in Ethiopia [33].

^c^NL = People who naturally emerge to take on leadership roles during the CLTS process [9].

^d^Kebele = The smallest administrative unit in Ethiopia [22].

^e^Andilaye = A WASH behaviour improvement intervention conducted in Ethiopia [21].

^f^RANAS = an acronym for Risks, Attitudes, Norms, Abilities, and Self-regulation; a theoretical model for understanding and influencing behaviour change [34].

^g^ODF = Faeces not exposed to the open air [9].

Abbreviations: WASH = Water, Sanitation and Hygiene (see Appendix 2 for details) CLTS = Community-Led Total Sanitation (see Appendix 2 for details).

Five studies reported infrastructure-related outcomes, such as latrine ownership or construction [23,25,28,30,32]. Four studies reported behavioural outcomes, including reductions in OD practices [20–22,24]. Three studies examined health-related outcomes, such as diarrhoea incidence or psychosocial stress [19,27,29]. Three studies included both behavioural and infrastructure-related outcomes [18,26,31].

### Intervention characteristics

As summarised in Table 2, most interventions were facilitated by NGOs or local community actors, and the duration commonly ranged from several months to approximately two years. Adaptations to the standard CLTS approach were frequently reported.

**Table 2.** Characteristics of interventions.

Author (Year)	Intervention facilitator	Implementation periods	Steps of the programme	Adaptations
Alemu et al (2023)	Health extension workers^a^ (HEWs)	After 2004	HEWs provide home-based health education and services for health promotion and disease prevention, including toilet promotion, handwashing education, and safe water handling, as well as community engagement and mobilisation.	The deployment of HEWs as part of HEP^b^. The HEP is a national programme but HEWs operate at the community level.
Alzúa et al (2019)	Direction Nationale de l’Assainissement^c^	September 2011–June 2012	Triggering, follow-up visits, awarding OD-free status	In Mali, the programme is implemented with support from UNICEF.
Cha et al (2021)	Officials from the district health office, health professionals from health centers, and HEWs.	January 2016–January 2017	CLTS triggering sessions	One to two CLTS promoters were selected from each intervention village to encourage toilet construction. In this study, the construction of improved toilets was promoted.
Crocker et al (2016a)	Plan (NGO) and other three NGOs (not specified)	November 2012–July 2013	CLTS: Three phases of CLTS pre-triggering, triggering event, post-triggering CLTS + Natural Leader^d^ (NL) training: NL were selected five months after the start of CLTS activities and were subsequently trained.	Following the CLTS Handbook guidelines. NL training: CLTS concepts, sanitation, handwashing, health impacts, latrine construction, and participatory techniques.
Crocker et al (2016b)	CLTS: HEWs Teacher-facilitated CLTS: Teacher	2012–2013	Three phases of CLTS pre-triggering, triggering event, post-triggering	In Ethiopia, special emphasis is placed on the practice of handwashing during the follow-up phase. Both groups of facilitators received training and monthly guidance from the NGO.
Freeman et al (2022)	Andilaye^e^: Government salaried officials from the Woreda^f^ Health Office, HEWs, and volunteer Women’s Development Army Leaders^g^ (WDALs).	September 2017–April 2018	Andilaye: starting with community mobilization and commitment events, followed by group dialogues with influential community members (community conversations), and then household counseling visits to caregivers, primarily mothers.	CLTS uses negative emotional motivators such as shame and disgust, whereas Andilaye promotes behaviour change through a positive approach. The Andilaye intervention focused on three WASH-related behavioural themes, informed by formative research: (1) sanitation, (2) personal hygiene, and (3) household environmental sanitation.
Harter et al (2018)	Pathfinder International (NGO)	2012–2014	Three phases of CLTS pre-triggering, triggering event, post-triggering	Following the CLTS Handbook guidelines. No adaptations described
Harter et al (2019a)	Global Communities (NGO)	July–December 2016	Three phases of CLTS pre-triggering, triggering event, post-triggering	Intervention protocols based on the CLTS Handbook. No adaptations described.
Harter et al (2019b)	Global Communities (NGO)	July–November 2016	Informative phase, triggering event, follow-up visits	Developed intervention protocols based on the CLTS Handbook.
Harter et al (2020)	NGO (not specified)	July–November 2016	Three phases of CLTS pre-triggering, triggering event, post-triggering	CLTS + RANAS^h^-Com: participants stepping up in front of the community after the triggering event and showing their commitment to construct latrines. CLTS + RANAS-Plan: In the week following the triggering event, all households in this intervention arm were visited, and a detailed action plan for latrine construction was developed. CLTS + RANAS-ComPlan: the two approaches combined.
Mlenga and Baraki (2015)	CLTS: Community Rural Health Motivators trained by IRD(NGO). SBSI: Government and NGO (not specified)	2012–2013	CLTS: Situation analysis, emotional triggering, development of an action plan SBSI: Training on latrine construction and provision of materials	No adaptations described.
Okolimong et al (2020)	Local leaders, community health workers, and other opinion leaders.	2011	Pre triggering, triggering, monitoring, and declaration of Open Defecation Free^i^ (ODF) status.	Community education was conducted twice a year through a radio programme.
Pickering et al (2015)	Programme staff employed by the government’s department of sanitation in the district of Koulikoro.	2011	CLTS triggering sessions	Latrine designs using locally available materials were encouraged.
Shiras et al (2018)	Water and Sanitation for the Urban Poor (WSUP)	March 2015–March 2016	Construction of pour-flush toilets with a septic tank connected to an infiltration pit (either squat or pedestal style), shared among compound residents.	Shared Latrine (SL): Designed for compounds with 20 or fewer residents. Compound Sanitation Block (CSB): Designed for compounds with more than 20 residents, with one cubicle per 20 people.
Tiwari et al (2017)	Government and NGO (not specified)	CLTS: August 2013–July 2015 Chiefdom orientations: May 2014–July 2015	CLTS: Triggering event Orientation: Chiefs are gathered and presented with the sanitation access status of their villages compared to neighboring chiefdoms. After orientation, sanitation targets are set.	CLTS: In Zambia, volunteer community champions are selected from among local residents to promote behaviour change within the community.

^a^HEWs = Government employees responsible for delivering health services at the community level within the HEP [33].

^b^HEP = A flagship programme in the health sector implemented in Ethiopia [33].

^c^Direction Nationale de l’Assainissement = French for National Sanitation Office [26].

^d^NL = People who naturally emerge to take on leadership roles during the CLTS process [9].

^e^Andilaye = A WASH behaviour improvement intervention conducted in Ethiopia [21].

^f^Woreda = Districts in Ethiopia [21].

^g^WDALs = Unpaid community health workers [21].

^h^RANAS = an acronym for Risks, Attitudes, Norms, Abilities, and Self-regulation; a theoretical model for understanding and influencing behaviour change [34].

^i^ODF = Faeces not exposed to the open air [9].

Abbreviations: WASH = Water, Sanitation and Hygiene (see Appendix 2 for details). CLTS = Community-Led Total Sanitation (see Appendix 2 for details). SBSI = Subsidy-Based Sanitation Interventions.

CLTS is characterised by the use of a triggering process to stimulate behaviour change, with most interventions structured around three phases described in the CLTS Handbook: pre-triggering, triggering, and post-triggering [9]. Some studies described adaptations, such as modifications to latrine construction materials and design [19,22,27,31,32]. In contrast, the Andilaye intervention, implemented by trained Woreda Health Office officials, Health Extension Workers; (HEWs), and volunteer Women’s Development Army Leaders (WDALs), aimed to promote behaviour change through a positive approach rather than relying on negative emotions such as shame or disgust typically associated with CLTS triggering [21]. HEWs mainly provided health education at the community level [18]. They promoted WASH behaviours through household visits and activities at health posts. Other interventions included shared toilets constructed at a ratio of one per 20 residents [29].

### Reported outcomes

Table 3 summarises the characteristics of reported outcomes, demonstrating considerable variation in both sample size and outcome domains.

**Table 3.** Characteristics of outcomes.

Author (Year)	Sample size (intervention)	Number of respondents (outcome measurement)	Respondent characteristics	Data collection method	Outcome measurement periods	Reported outcomes
Alemu et al (2023)	7,122 households in 186 kebeles	6,430 households	Female	Interview and on-site observations	March 2018–May 2019	72.7% of households had at least one latrine—29.2% of which were improved facilities – while 27.3% practiced open defecation (OD). Exposure to health education delivered by HEWs^a^ was positively and significantly associated with latrine availability (AOR = 1.44, 95% CI = 1.15–1.80), but showed a significant negative association with latrine use (AOR = 0.62, 95% CI = 0.44–0.89).
Alzúa et al (2019)	402 villages	5,206 households	Households with children under the age of 10	Interview and on-site observations	April–June 2014	CLTS increased latrine ownership by 29% (62% vs. 33%) and reduced OD to 40% (vs. 82%), including among children (41% vs. 82%). Both are statistically significant. (*p* < 0.01)
Cha et al (2021)	Case: 1,737 households Control: 1,795 households in 48 villages	Case: 455 households Control: 451 households	Caregiver or households with children under the age of 5	Caregivers recorded the duration of diarrhoea using a diary format.	June 2016–January 2017	During 140 days, diarrhoea incidence and prevalence were lower in the intervention group, with an incidence ratio of 0.66 (*p* = 0.03) and a prevalence ratio of 0.70 (*p* = 0.02), while there was no difference in duration. (*p* = 0.48). In the intervention group, the 7-day prevalence of diarrhoea decreased from 22.2% at baseline to 11.8% at 3 months (*p* = 0.04) and further to 7.7% at 10 months; however, the latter reduction was not statistically significant.
Crocker et al (2016a)	1,759 households	1,708 households	N/A	Interview and on-site observations	May 2014	Villages with CLTS + NL^b^ training saw a 19.9% point drop in OD, compared to villages that received only CLTS (*p* < 0.001), with shared latrine use up 4.3% points (*p* = 0.001) and private latrine use up 18.3% points (*p* < 0.001).
Crocker et al (2016b)	2,444 households in 75 villages	Baseline: 2,182 households Follow-up: 2,263 households	N/A	Interview	October 2013	OD fell in both groups, with HEWs-led CLTS reducing it by 22% and teacher-facilitated CLTS by 13.8%, an 8.2% point difference (*p* = 0.048).
Freeman et al (2022)	1,500 households	Case: 743 households Control: 729 households	Female primary caregiver (90%)/ Households with at least one child aged 1–9 years	Interview and on-site observations	March-May 2019	Andilaye^c^ intervention did not increase latrine access. There was no statistically significant difference in the prevalence of improved latrines (PR = 1.13; 95% CI = 0.81–1.59) or in fully constructed latrines (PR = 1.15; 95% CI = 0.86–1.54) between case and control. The intervention did not have a statistically significant impact on defecation practices. (PR = 1.05; 95% CI = 0.76–1.45)
Harter et al (2018)	640 households in 32 communities	603 cases	Primary caregiver	Self-reported questionnaires and on-site observations	November 2014 (at least 8 months after CLTS)	Latrine ownership status A) Households which participated in CLTS = 79% B) Households that missed CLTS sessions but received information indirectly from relatives, friends, or neighbors = 76% C) Households living in communities that underwent a CLTS triggering event but did not receive any CLTS-related information = 57% D) Households living in the control communities, where CLTS was not performed = 28% *p* value not reported.
Harter et al (2019a)	25 households in each of 102 communities	1,877 households	Over 18 years and resident in the community for at least three months.	Interview	March–April 2017	Overall, community latrine coverage increased pre-post by 67.6%. *p* value not reported.
Harter et al (2019b)	3,216 households	2,607 households	Over 18 years olds	Interview and on-site observations	February–March 2018	At follow-up, OD was 46.4% in the CLTS group and 88.4% in the control group. (*p* < 0.001, OR = 0.09, 95% CI = 0.05–0.17)
Harter et al (2020)	3,216 households	3,216 households	Over 18 years and resident in the community for at least three months	Interview	First follow-up survey: February–March 2017 Second follow-up survey: February–March 2018	Latrine coverage rose from 3.2% at baseline to 68.2% after the intervention. Latrine construction: (1)CLTS alone = 65.5% (2)CLTS+RANAS-Com^d^ = 73.2% (3)CLTS+RANAS-Plan^e^ = 67.1% (4)CLTS+RANAS-ComPlan^f^ = 67.7% Control communities: 7.8% There was a significant difference in latrine ownership between the CLTS and control groups (*p* < 0.001), but no significant difference was found between the CLTS-only and CLTS+RANAS groups. (2)*p* = 0.597, (3)*p* = 0.964, (4)*p* = 0.962
Mlenga and Baraki (2015)	Case:100 households Control:100 households	200 households	N/A	Knowledge, Attitudes, and Practices (KAP) survey conducted by trained enumerators	2013–2014 (within 10–16 months after the project started)	CLTS: At project start, 70% lacked latrines and practiced OD; 10–16 months later, this dropped by 40%. SBSI: After hardware support, latrine ownership rose to 90%, but 40% lacked key materials, and only 54% were fully functional. *p* value not reported.
Okolimong et al (2020)	Case 200 households Control: 200 households	Case 200 households Control: 200 households	Over 18 years old	Interview	May 2018	Overall, 35% of case communities and 6% of control communities achieved Open Defecation Free^g^ (ODF) status, and the difference was statistically significant (*p* < 0.001). Latrine coverage was 97.5% in the CLTS intervention areas and 91.5% in the non-intervention areas, and this difference was not statistically significant. (*p* = 0.100)
Pickering et al (2015)	Case: 2,365 households in 60 villages Control: 2,167 households in 61 villages	Case: 2,120 households Control: 1,911 households	Female primary caregiver of the youngest child in the household/ Households with at least one child < 10 years	Interview	March–May 2013	There was no significant difference in the prevalence of diarrhoea between case and control. 2 days-recall: (PR = 0.93, 95% CI = 0.76–1.14, *p* = 0.486) 2 week-recall (PR = 0.98, 95% CI = 0.82–1.17, *p* = 0.787)
Shiras et al (2018)	11 bairros (neighborhoods)	96 participants (70 women and 26 men)	Over 18 years olds Individuals were grouped as: with children under 3, youngest child 3–18, no children or youngest over 18, married within 2 years, and moved to the compound within 2 years.	Interview, focus group discussions and on-site observations	Within a year of the programme	Sanitation-related distress includes safety and privacy issues, disgust or shame about latrines, and management conflicts. Shared latrine users reported fewer safety concerns (41%) than Compound Sanitation Block (CSB) and Traditional Latrine (TL) users (~80%), while management stress was highest among CSB (77%) and TL users (65%). *p* value not reported.
Tiwari et al (2017)	10,932 villages	10,932 villages	N/A	Mobile-to-web health information management system (District Health Information System 2, DHIS2)	August 2013–July 2015	Of 10,932 villages, 3,137 achieved universal latrine access, with chiefdom orientations linked to a 30.4% increase in household latrine access (*p* < 0.0001).

^a^HEWs = Health Extension Workers, Government employees responsible for delivering health services at the community level within the HEP [33].

^b^NL = Natural Leader, People who naturally emerge to take on leadership roles during the CLTS process [9].

^c^Andilaye = A WASH behaviour improvement intervention conducted in Ethiopia [21].

^d^RANAS-Com = Public commitment to build latrines.

^e^RANAS-Plan = Household visits to make latrine action plans.

^f^RANAS-ComPlan = Combination of RANAS-Com and RANAS-Plan.

^g^Open Defecation Free = Faeces not exposed to the open air [9].

Abbreviations: WASH = Water, Sanitation and Hygiene (see Appendix 2 for details). CLTS = Community-Led Total Sanitation (see Appendix 2 for details). SBSI = Subsidy-Based Sanitation Interventions PR = Prevalence Ratio.

Overall, 12 studies reported improvements in the assessed outcomes [19,20,22–26,28–32], while three studies reported no significant improvement or mixed findings [18,21,27]. Among the eight studies assessing the effects of CLTS-only interventions [19,22–24,26–28,31], four reported outcomes related to latrine use [23,26,28,31]. Alzua et al. reported a statistically significant increase of 29% in latrine ownership rates compared to the control group [26], while Harter et al. reported increases of 48–51% compared to the control group [28]. Harter et al. also reported a 67.6% increase in latrine coverage compared to the pre-intervention level [23]. In contrast, Okolimong et al. reported no statistically significant difference in latrine ownership between the intervention and non-intervention groups [31]. Regarding the rate of OD practice, four studies reported results [22,24,26,31], two of which also reported on latrine use [26,31]. Alzua et al. reported a 40% reduction [26], Okolimong et al. reported a 23% reduction [31], and Harter et al. reported a 42% reduction compared to the control group [24], all of which were statistically significant. Crocker et al. evaluated CLTS led by teachers and CLTS led by HEWs, with the HEWs-led intervention achieving an 8.2% greater reduction in OD (22% vs 13.8%) [22], which was statistically significant.

Two CLTS studies reported on diarrhoea among children [19,27], with inconsistent outcomes between studies. Cha et al. reported statistically significant reductions in diarrhoea incidence and prevalence over a 140-day period; however, no difference was observed in duration [19]. In contrast, Pickering et al. found no statistically significant difference in diarrhoea prevalence between the intervention and control groups across both 2-day and 2-week recall periods [27].

Among the four studies comparing CLTS with other intervention approaches [20,25,30,32], Mlenga and Baraki reported that latrine ownership was approximately 30% higher under SBSI compared to CLTS. However, 40% of latrine owners under SBSI faced material shortages [30]. Three of these studies compared CLTS alone with CLTS combined with additional components [20,25,32]. Harter et al. found that adding a RANAS-based intervention (Risks, Attitudes, Norms, Abilities, and Self-regulation) [34] to CLTS resulted in only a 2–8% increase in latrine coverage compared to CLTS alone, with no statistically significant difference [25]. Crocker et al. reported that training NL as part of CLTS significantly reduced the OD rate by 19.9% compared to CLTS alone [20]. Tiwari et al. found that integrating chiefdom orientation with CLTS statistically significantly increased latrine access by 30.4% [32].

Looking at the three interventions other than CLTS [18,21,29], Freeman et al. (2022) reported that the Andilaye programme did not lead to statistically significant changes in latrine access or defecation practices [21]. Shiras et al. (2018) found that a shared sanitation intervention reduced stress related to safety and privacy [29]. Alemu et al. (2023) reported that in a health education intervention delivered by HEWs, latrine use was significantly negatively associated with exposure to health education [18].

Except for two studies reporting at the individual and village level [29,32], the sample sizes in the remaining 13 studies were reported at the household level, ranging from under 1,000 households [26,28,30,31] to more than 4,000 households [18,27].

### Barriers and facilitators

Table 4 summarises the reported barriers and facilitators, which were reported in 10 studies [21–24,26,28–32], although the specific content varied across studies. Four studies reported both barriers and facilitators [21,22,26,29], two studies reported only barriers [30,31], and four studies reported only facilitators [23,24,28,32].

**Table 4.** Intervention barriers and facilitators.

Author (Year)	Reported barriers	Reported facilitators
Alzúa et al (2019)	While CLTS led to latrine construction, facilities were often inadequate, or households were reluctant to fully meet CLTS standards.	Many households joined triggering activities with their children, and child-focused activities were organised.
Crocker et al (2016b)	In teacher-led villages, collaboration with influential kebele^a^ leaders was limited.	In HEWs^b^-led villages, existing ties with the kebele contributed to higher community participation in triggering events.
Freeman et al (2022)	Intervention was not implemented as originally designed due to low participation in community events and insufficient delivery of household counselling visits.	The intervention was theory-informed and designed with feedback from stakeholders across community and regional levels.
Harter et al (2018)	N/A	People who are in a relationship, have more years of schooling, or live in low flood-risk areas are more likely to own a latrine. Strong social ties (trust, solidarity, and inclusion) also increase latrine ownership, showing that both personal and community factors play key roles.
Harter et al (2019a)	N/A	Incentives, follow-up visits, natural leaders^c^ (NL), and community participation in the triggering event have a significant impact on latrine coverage.
Harter et al (2019b)	N/A	Communities with higher social identification had greater success.
Mlenga and Baraki (2015)	CLTS effectiveness varies depending on facilitator skills. The main reasons for not having a latrine are lack of construction materials and lack of funds.	N/A
Okolimong et al (2020)	The use of local materials increases latrine coverage but is often associated with poor construction quality.	N/A
Shiras et al (2018)	Poor latrine management (e.g. lack of cleaning) is a source of stress, leading to neighbour conflicts and social exclusion.	Latrines equipped with doors and locks and situated close within the compound were perceived by many users to enhance their sense of security.
Tiwari et al (2017)	N/A	Deepening chiefs’ knowledge of CLTS raised Community Champions’ status and improved sanitation access. Smaller villages were more likely to reach universal coverage than larger villages (reason not specified).

^a^Kebele = The smallest administrative unit in Ethiopia [22].

^b^HEWs = Health Extension Workers, Government employees responsible for delivering health services at the community level within the HEP [33].

^c^NL = Natural Leader, People who naturally emerge to take on leadership roles during CLTS [9].

Abbreviations: CLTS = Community-Led Total Sanitation (see Appendix 2 for details).

Regarding barriers, the lack of effectiveness of the Andilaye intervention (even though it was robustly based on theoretical frameworks) was explained by authors as being due to it not being implemented as planned [21]. With regard to shared latrines, while doors and locks contributed to a sense of safety, issues related to maintenance, such as cleaning, were reported as sources of stress and conflict among residents [29].

Studies that implemented CLTS reported on barriers such as limited skills of facilitators during implementation [30] and insufficient quality or design of latrines [26,31]. On the other hand, a comparison of CLTS implemented by teachers and HEWs reported that the weak relationship between teachers and community leaders acted as a barrier, whereas the ongoing connection between HEWs and the community served as a facilitator [22].

Other reported facilitators in CLTS studies included the absence of flooding in the intervention area and smaller village size – these were often associated with higher latrine ownership [28,32]. Additionally, strong social relationships within the community and a high level of social identification were also reported as important facilitators [24,28].

## Discussion

In this scoping review, 15 community-based interventions aimed at eliminating OD were identified. Of these, eight studies implemented CLTS alone [19,22–24,26–28], three implemented CLTS with additional components [20,25,32], one study compared CLTS with SBSI [30], and three involved other interventions [18,21,29]. With the exception of three studies [18,21,27], the interventions generally reported improvements in the assessed outcomes [19,20,22–26,28–32]. Ten studies reported barriers, facilitators, or both [21–24,26,28–32].

CLTS was the most frequently implemented approach among the studies included. Although the steps and processes were explained in the studies included, it is unclear whether all of them were fully followed by the implementers. Additionally, follow-up periods after CLTS implementation varied from less than one year to seven years, which may have influenced the observed outcomes.

Among eight outcomes from studies implementing CLTS alone [19,22–24,26–28,31], seven reported improvements in toilet ownership or reductions in OD [19,22–24,26,28,31]. Pickering et al. (2015), however, found no significant difference in child diarrhoea prevalence between intervention and control groups [27]. Environmental differences across countries and regions, as well as community heterogeneity, mean that the success of CLTS depends on context, with smaller communities and fewer households generally showing better outcomes [35]. Cha et al. reported reductions in diarrhoea prevalence, while this study used caregiver-maintained diaries [19], Pickering et al. relied on caregiver self-reports, which may have introduced recall bias [27].

Harter et al. reported that relationships and connections within a community can facilitate the implementation of interventions [24,28]. However, strong social cohesion could also sometimes lead to the abandonment of CLTS. For instance, in Burkina Faso, shared cultural practices such as burial customs and beliefs supporting OD led the community as a whole to abandon the CLTS process [36]. This suggests that the implementation of CLTS requires not only consideration of physical and contextual factors but also alignment with the cultural and religious practices of each country and region [23,36].

Three studies examined CLTS in combination with additional components: NL training [20], RANAS-based intervention [25], and chiefdom orientation [32]. Interventions combining CLTS with either NL training or chiefdom orientation resulted in reductions in OD and increases in latrine ownership, both of which were statistically significant compared with CLTS alone. In contrast, the combination of CLTS with the RANAS-based intervention led to a statistically significant increase in latrine ownership compared with the control group, but no significant difference was observed compared with CLTS alone. This indicates that the addition of the RANAS-based intervention did not yield further improvements compared with CLTS alone.

In addition to Crocker et al. [20], Kapatuka has also reported that training NL is crucial for achieving and sustaining Open Defecation Free (ODF) status [37]. Crocker et al. showed that strengthening NL’ knowledge and skills improved the dissemination of CLTS messages [20], while Kapatuka highlighted the role of enhanced follow-up activities, such as household visits, in maintaining ODF [37]. These findings suggest that enhancing NL capacity, tailored to specific objectives and stages of intervention, may lead to further improvements in CLTS outcomes.

Overall, while combining CLTS with other elements does not necessarily guarantee greater effectiveness than CLTS alone, when designed with consideration of local objectives and context such combinations may further enhance the success of CLTS interventions.

Standalone interventions other than CLTS included Health education [18], Andilaye [21], and shared latrine construction [29], with two of these showing limited or mixed findings [18,21]. In the case of Health education [18], instruction by HEWs was positively associated with latrine ownership but negatively associated with latrine use. This finding is based on cross-sectional data and should not be interpreted as evidence of a causal relationship. It may instead reflect underlying contextual or behavioural factors not investigated in the study. For the Andilaye approach [21], no statistically significant effects were observed on latrine ownership or OD behaviour. Although HEWs and WDALs received training and workshops, only approximately 43% of households reported receiving home visits, suggesting that the actions of those providing knowledge can influence intervention outcomes. In Mozambique’s informal slums, shared latrine construction was evaluated differently, with psychosocial stress measured as an outcome [29]. While improvements in safety and privacy were observed, challenges related to cleaning and management were reported, which could lead to latrine non-use and OD. These findings highlight the importance of achieving consensus among residents regarding latrine management.

In contrast with this review, Garn et al. reported that sanitation interventions other than CLTS showed larger effect sizes in improving latrine coverage [38]. However, both reviews are consistent in indicating that CLTS contributes to improvements in latrine coverage. This apparent discrepancy may be partly explained by differences in review objectives and methodological approaches, as Garn et al. focused on effect size estimates derived from meta-analysis, whereas the present scoping review synthesised evidence based on the frequency of reported effects and contextual effectiveness. In contrast, the findings of this review are largely consistent with those of Venkataramanan et al., who concluded that CLTS is associated with increased latrine ownership and reductions in open defecation, while evidence on sanitation-related health outcomes remains limited and inconsistent [16]. Also similar to this review, Venkataramanan et al. highlighted that the number of studies assessing health outcomes was small and that findings varied across settings [16]. In line with the review by Venkataramanan et al., this review suggests that variation in CLTS outcomes may be influenced by implementation-related factors, such as facilitator skills and follow-up, as well as by community context and participation patterns. Taken together, these findings suggest that CLTS outcomes may vary across settings, with implementation-related factors and community context frequently highlighted in the literature.

### Strengths and limitations

This review focused on studies mentioning ‘open defecation’ or its variants in the title or abstract to maintain a clear focus on interventions explicitly targeting OD practices. However, relevant studies without these terms might have been missed. Manual screening helped to identify one such study, but the possibility of having missed other potentially relevant articles cannot be excluded.

In addition, this review searched Ovid MEDLINE, Web of Science, and EBSCO CINAHL, which provide broad coverage of biomedical, public health, global health, nursing, and interdisciplinary literature. Inclusion of other databases and search platforms such as Global Health, Embase, and Scopus, could have potentially identified additional relevant studies.

Studies focusing solely on intervention sustainability or cost-effectiveness were also excluded, as the primary aim of this review was to map the types of community-based interventions targeting open defecation and their reported outcomes. While sustainability and cost-effectiveness are important considerations – particularly in relation to ODF relapse – they address different analytical questions and were therefore considered to be beyond the scope of this review. Nevertheless, sustainability remains a recognised challenge in the literature [39]. Furthermore, in studies measuring multiple sanitation outcomes, only the main results related to OD were extracted, which may limit the fairness of comparisons. Variation in the unit of analysis across studies (household, individual, or village level) may further constrain comparability, as outcomes measured at different levels capture distinct dimensions of intervention effects. In addition, although multiple study designs, including RCTs, were employed in the included studies, differences in findings by study design were not examined in this review. The strength of evidence may vary depending on the study design (observational studies, for example, only show associations and are not appropriate to investigate causal relationships). A more robust comparison of the effectiveness across different interventions (and different study designs) would require a systematic review.

Although the interventions primarily targeted school-age individuals and adults, some studies assessed health outcomes among younger children to capture indirect household-level effects. This reflects differences in outcome measurement rather than differences in the target population of the interventions.

Finally, most included studies were conducted in Ethiopia and Ghana, which may limit the generalisability of the findings to other Sub-Saharan African contexts with different health systems, sociocultural, demographic, or policy environments.

Review strengths include the use of well-established scoping review guidance, independent screening, and comprehensive search strategies for three databases. As the aim was to map the scope of evidence, different community-based interventions were eligible for inclusion, and only studies with clearly described intervention components were extracted. This allowed for a comprehensive understanding of intervention approaches. Additionally, data on barriers and facilitators provide useful information for designing future interventions.

### Implications for public health practice and research

Detailed reporting of intervention implementation can help identify barriers and areas for improvement. Considering sustained behaviour change is important; further studies should investigate key factors influencing sustainability of community-based interventions targeting OD practices.

## Conclusion

In SSA, CLTS was the most frequently implemented community-based intervention to eliminate OD. While often implemented alone, some interventions combined it with other components. Other standalone interventions were less common and generally reported fewer improvements in the assessed outcomes. Designing interventions to suit the local context and engaging community leaders may enhance effectiveness. Future efforts should focus on long-term evaluation and sustaining behaviour change.

## Supplementary Material

PRISMA ScR checklist.docx

Appendices Global Health Action AM 24032026 clean.docx

## Data Availability

The authors confirm that the data supporting the findings of this study are available within the article [and/or] its supplementary materials.
