# Supplementary material for: Community-based interventions addressing open defecation in Sub-Saharan Africa: a scoping review
Source: Glob Health Action. 2026 Apr 13;19(1):2655033. doi: 10.1080/16549716.2026.2655033 (PMC13072696; doi:10.1080/16549716.2026.2655033)
Supplement: Appendices Global Health Action AM 24032026 clean.docx [file ZGHA_A_2655033_SM0742.docx]

Appendix 1: Search Strategy

This appendix presents the detailed search strategies used for each three databases in this review.

Table 1: Search strategy for MEDLINE (Epub Ahead of Print, In-Process, In-Data-Review & Other Non-Indexed Citations, Daily, and Versions <1946 to June 26, 2025>)

| # | Query | Results |
| --- | --- | --- |
| 1 | exp Africa South of the Sahara/ OR exp Africa, Central/ OR exp Africa, Eastern/ OR exp Africa, Southern/ OR exp Africa, Western/ | 284,880 |
| 2 | (SSA OR Sub-Saharan Africa OR Subsaharan Africa OR Central Africa OR East* Africa OR West* Africa OR Southern Africa OR Middle Africa).tw. | 69,378 |
| 3 | ((East adj1 Africa) OR (West adj1 Africa) OR (South adj1 Africa)).tw. | 66,808 |
| 4 | (British Indian Ocean Territory OR Burundi OR Comoros OR Djibouti OR Eritrea OR Ethiopia OR French Southern Territories OR Kenya OR Madagascar OR Malawi OR Mauritius OR Mayotte OR Mozambique OR Reunion OR Rwanda OR Seychelles OR Somalia OR South Sudan OR Sudan OR Uganda OR United Republic of Tanzania OR Tanzania OR Zambia OR Zimbabwe OR Angola OR Cameroon OR Central African Republic OR Central Africa OR Chad OR Congo OR Democratic Republic of the Congo OR DRC OR DR Congo OR Gabon OR (Sao Tome and Principe) OR Botswana OR Eswatini OR Lesotho OR Namibia OR South Africa OR Benin OR Burkina Faso OR Cabo Verde OR Cape Verde OR Cote d'Ivoire OR Ivory Coast OR Gambia OR Ghana OR Guinea OR Equatorial Guinea OR Guinea-Bissau OR Liberia OR Mali OR Mauritania OR Niger OR Nigeria OR Saint Helena OR Senegal OR Sierra Leone OR Togo).tw. | 435,353 |
| 5 | 1 OR 2 OR 3 OR 4 | 539,383 |
| 6 | (open defecation OR (open adj2 defec*)).tw. | 1,195 |
| 7 | (Latrine us* OR Latrine Coverage OR Sanitary Latrine OR Toilet Facilit* OR sanitation facilit*).tw. | 1,663 |
| 8 | Toilet Facilities/ | 2,047 |
| 9 | (community-led OR community led OR community-based OR community based OR community-level OR community level OR community approach OR school*).tw. | 474,847 |
| 10 | Schools/ | 56,596 |
| 11 | (Community-Led Total Sanitation OR CLTS OR Community Approaches to Total Sanitation OR CATS OR Sanitation Intervention OR Behavio?r* Change Communication OR WASH Intervention* OR WASH behavio?r* OR RANAS OR (Social and Behavio?r Change Communication) OR SBCC OR sanitation marketing OR nudging OR nudge).tw. | 75,163 |
| 12 | 7 OR 8 OR 9 OR 10 OR 11 | 558,695 |
| 13 | 5 AND 6 AND 12 | 202 |

Table 2: Search strategy for Web of Science

| # | Query | Results |
| --- | --- | --- |
| 1 | (('open defecation' OR (open N2 defec*))) (Title) OR (('open defecation' OR (open N2 defec*))) (Abstract) | 1,839 |
| 2 | SSA OR ‘Sub-Saharan Africa’ OR ‘SubsaharanAfrica’ OR ‘Central Africa’ OR ‘East* Africa’ OR ‘West* Africa’ OR ‘Southern Africa’ OR ‘Middle Africa’ (Title) OR SSA OR ‘Sub-Saharan Africa’ OR ‘SubsaharanAfrica’ OR ‘Central Africa’ OR ‘East* Africa’ OR ‘West* Africa’ OR ‘Southern Africa’ OR ‘Middle Africa’ (Abstract) | 209,871 |
| 3 | (((East N1 Africa) OR (West N1 Africa) OR(South N1 Africa))) (Title) OR (((East N1 Africa) OR (West N1 Africa) OR(South N1 Africa))) (Abstract) | 57 |
| 4 | TI= ((British Indian Ocean Territory OR Burundi OR Comoros OR Djibouti OR Eritrea OR Ethiopia OR French Southern Territories OR Kenya OR Madagascar OR Malawi OR Mauritius OR Mayotte OR Mozambique OR Reunion OR Rwanda OR Seychelles OR Somalia OR South Sudan OR Sudan OR Uganda OR United Republic of Tanzania OR Tanzania OR Zambia OR Zimbabwe OR Angola OR Cameroon OR Central African Republic OR Central Africa OR Chad OR Congo OR Democratic Republic of the Congo OR DRC OR DR Congo OR Gabon OR (Sao Tome and Principe) OR Botswana OR Eswatini OR Lesotho OR Namibia OR South Africa OR Benin OR Burkina Faso OR Cabo Verde OR Cape Verde OR Cote d'Ivoire OR Ivory Coast OR Gambia OR Ghana OR Guinea OR Equatorial Guinea OR Guinea-Bissau OR Liberia OR Mali OR Mauritania OR Niger OR Nigeria OR Saint Helena OR Senegal OR Sierra Leone OR Togo)) OR AB=((British Indian Ocean Territory OR Burundi OR Comoros OR Djibouti OR Eritrea OR Ethiopia OR French Southern Territories OR Kenya OR Madagascar OR Malawi OR Mauritius OR Mayotte OR Mozambique OR Reunion OR Rwanda OR Seychelles OR Somalia OR South Sudan OR Sudan OR Uganda OR United Republic of Tanzania OR Tanzania OR Zambia OR Zimbabwe OR Angola OR Cameroon OR Central African Republic OR Central Africa OR Chad OR Congo OR Democratic Republic of the Congo OR DRC OR DR Congo OR Gabon OR (Sao Tome and Principe) OR Botswana OR Eswatini OR Lesotho OR Namibia OR South Africa OR Benin OR Burkina Faso OR Cabo Verde OR Cape Verde OR Cote d'Ivoire OR Ivory Coast OR Gambia OR Ghana OR Guinea OR Equatorial Guinea OR Guinea-Bissau OR Liberia OR Mali OR Mauritania OR Niger OR Nigeria OR Saint Helena OR Senegal OR Sierra Leone OR Togo)) | 881,147 |
| 5 | #2 OR #3 OR #4 | 976,163 |
| 6 | (('Latrine us*' OR 'Latrine Coverage' OR 'Sanitary Latrine' OR 'Toilet Facilit*' OR 'sanitation facilit*')) (Title) OR (('Latrine us*' OR 'Latrine Coverage' OR 'Sanitary Latrine' OR 'Toilet Facilit*' OR 'sanitation facilit*')) (Abstract) | 7,016 |
| 7 | (('community-led' OR 'community led' OR 'community-based' OR 'community based' OR 'community-level' OR 'community level' OR 'community approach' OR school*)) (Title) OR (('community-led' OR 'community led' OR 'community-based' OR 'community based' OR 'community-level' OR 'community level' OR 'community approach' OR school*)) (Abstract) | No data available due to error when saving search results from database |
| 8 | ‘Community-Led Total Sanitation’ OR CLTS OR ‘Community Approaches to Total Sanitation’ OR CATS OR ‘Sanitation Intervention’ OR ‘Behavio?r* Change Communication’ OR ‘WASH Intervention*’ OR ‘WASH behavio?r*’ OR RANAS OR SBCC OR ‘sanitation marketing’ OR nudging OR nudge (Title) OR ‘Community-Led Total Sanitation’ OR CLTS OR ‘Community Approaches to Total Sanitation’ OR CATS OR ‘Sanitation Intervention’ OR ‘Behavio?r* Change Communication’ OR ‘WASH Intervention*’ OR ‘WASH behavio?r*’ OR RANAS OR SBCC OR ‘sanitation marketing’ OR nudging OR nudge (Abstract) | 275,042 |
| 9 | #6 OR #7 OR #8 | 1,980,561 |
| 10 | #1 AND #5 AND #9 | 288 |

Table 3: Search strategy for EBSCO CINAHL

|  | Query | Results |
| --- | --- | --- |
| S1 | XB (SSA OR Sub Saharan Africa OR Subsaharan Africa OR Central Africa OR East* Africa OR West* Africa OR Southern Africa OR Middle Africa) | 17,519 |
| S2 | XB (((((East N1 Africa) OR (West N1 Africa) OR (South N1 Africa)))) | 19,639 |
| S3 | (MH "Africa South of the Sahara") OR (MH "Africa, Southern") OR (MH "Africa, Western") OR (MH "Africa, Eastern") OR (MH "Africa, Central") | 6,697 |
| S4 | XB (((British Indian Ocean Territory OR Burundi OR Comoros OR Djibouti OR Eritrea OR Ethiopia OR French Southern Territories OR Kenya OR Madagascar OR Malawi OR Mauritius OR Mayotte OR Mozambique OR Reunion OR Rwanda OR Seychelles OR Somalia OR South Sudan OR Sudan OR Uganda OR United Republic of Tanzania OR Tanzania OR Zambia OR Zimbabwe OR Angola OR Cameroon OR Central African Republic OR Central Africa OR Chad OR Congo OR Democratic Republic of the Congo OR DRC OR DR Congo OR Gabon OR (Sao Tome and Principe) OR Botswana OR Eswatini OR Lesotho OR Namibia OR South Africa OR Benin OR Burkina Faso OR Cabo Verde OR Cape Verde OR Cote d'Ivoire OR Ivory Coast OR Gambia OR Ghana OR Guinea OR Equatorial Guinea OR Guinea-Bissau OR Liberia OR Mali OR Mauritania OR Niger OR Nigeria OR Saint Helena OR Senegal OR Sierra Leone OR Togo))) | 88,989 |
| S5 | S1 OR S2 OR S3 OR S4 | 99,255 |
| S6 | XB ((open defecation OR (open N2 defec*))) | 412 |
| S7 | XB (Latrine us* OR Latrine Coverage OR Sanitary Latrine OR Toilet Facilit* OR sanitation facilit*)) | 686 |
| S8 | MH "Toilet Facilities" | 1,083 |
| S9 | XB ((Community-led OR Community led OR Community-based OR Community based OR Community-level OR Community level OR Community approach OR school*) | 239,245 |
| S10 | (MH "Schools, Elementary") OR (MH "Schools, Middle") OR (MH "Schools, Secondary") | 13,575 |
| S11 | XB (((Community-Led Total Sanitation OR CLTS OR Community Approaches to Total Sanitation OR CATS OR Sanitation Intervention OR Behavio?r* Change Communication OR WASH Intervention* OR WASH behavio?r* OR RANAS OR (Social and Behavio?r Change Communication) OR SBCC OR sanitation marketing OR nudging OR nudge))) | 10,058 |
| S12 | S7 OR S8 OR S9 OR S10 OR S11 | 252,808 |
| S13 | S5 AND S6 AND S12 | 71 |

Appendix 2: Glossary of Terms

CLTS (Community-Led Total Sanitation)

An integrated approach aimed at eliminating and sustaining the end of open defecation. Since 2000, it has spread globally, starting in Bangladesh. It is characterized by a “trigger” that encourages community members to review their sanitation practices and initiate behaviour change themselves. The sequence of steps consists of Pre-triggering (Selecting a community, Introduction and building rapport), Triggering (Participatory sanitation profile analysis, Ignition moment), and Post-triggering (Action planning by the community, Follow up), and can be modified or adjusted according to the situation.^1^

WASH (Water, Sanitation, and Hygiene)

Safe drinking water, sanitation facilities, and hygiene practices. Essential for maintaining health and preventing diarrheal and neglected tropical diseases. Access to safe water and proper sanitation services significantly reduces disease risk and mortality.^2^

References

1. Kar K, Chambers R. Handbook on Community-Led Total Sanitation [Internet]. Plan International (UK); 2008 Mar [cited 2025 Aug 14]. Available from: <https://plan-international.org/publications/handbook-on-community-led-total-sanitation/>

2. World Health Organization. Water, sanitation and hygiene (WASH) [Internet]. Geneva: WHO; [cited 2025 Aug 17]. Available from: <https://www.who.int/health-topics/water-sanitation-and-hygiene-wash#tab=tab_1>
